# Can we keep neck breathers safe? A survey on training adequacy of medical staff caring for tracheostomy/laryngectomy patients

**DOI:** 10.1186/cc12106

**Published:** 2013-03-19

**Authors:** C Tjen, G Rajendran, S Hutchinson

**Affiliations:** 1 Norfolk and Norwich University Hospital, Norwich, UK

## Introduction

A fatal incident related to a blocked tracheostomy tube prompted a review in our Trust. To provide safe tracheostomy care, changes in staffing, education and operational policies were recommended. Training of potential first responders to tracheostomy or laryngectomy emergencies remains outstanding. We aim to quantify the training deficit. Tracheostomies are common in critical care but these patients require ongoing management of an artificial airway on discharge to the ward and even the community. In 2010 our critical care unit cared for 108 tracheostomy patients, of which 30 were transferred to the wards. The 4th National Audit Project highlighted complications including hypoxic brain injury and death [1] and the National Patient Safety Agency recognised a number of avoidable aspects [2]. Existing guidelines for management of these patients including emergencies are not widely known.

## Methods

An anonymous online survey was sent to all trainees who may respond to a tracheostomy emergency in our organisation. Trainees in anaesthesia/critical care, general medicine, general surgery, ENT, thoracics and A&E were approached. All completed forms were included.

## Results

We achieved a response rate of 39% (65/168). Respondents comprised: 33% anaesthesia/critical care, 47% medicine and 14% surgery. Over one-half (36/65) had managed tracheostomy/laryngectomy emergencies, with 42% (15/36) of these incidents occurring on the wards and one in an outpatient clinic. Only 20% (13/65) had received any formal training on management of a blocked/misplaced tracheostomy tube and only 18% (12/65) were aware of any guidelines. One-third of responders lacked confidence in management of these emergencies and 88% felt they would benefit from formal training including simulation.

## Conclusion

The population of patients with exteriorised tracheas is increasing and represents a high-risk group. Management of airway emergencies in these patients is not part of standard life-support courses. According to our trainees, these scenarios are relatively common and a significant proportion of first responders are poorly equipped to deal with them. Our Trust will be including specific training on the emergency management of neck breathers as part of in-house resuscitation training. We would contend that national resuscitation courses should consider doing the same.
